# The Effects of NMDA Subunit Composition on Calcium Influx and Spike Timing-Dependent Plasticity in Striatal Medium Spiny Neurons

**DOI:** 10.1371/journal.pcbi.1002493

**Published:** 2012-04-19

**Authors:** Rebekah C. Evans, Teresa Morera-Herreras, Yihui Cui, Kai Du, Tom Sheehan, Jeanette Hellgren Kotaleski, Laurent Venance, Kim T. Blackwell

**Affiliations:** 1 George Mason University, The Krasnow Institute for Advanced Study, MS 2A1, Fairfax, Virginia, United States of America; 2 Center for Interdisciplinary Research in Biology, CNRS 7241/INSERM U1050, Collège de France, Paris, France; 3 Department of Neuroscience, Karolinska Institute, Stockholm, Sweden; University of Freiburg, Germany

## Abstract

Calcium through NMDA receptors (NMDARs) is necessary for the long-term potentiation (LTP) of synaptic strength; however, NMDARs differ in several properties that can influence the amount of calcium influx into the spine. These properties, such as sensitivity to magnesium block and conductance decay kinetics, change the receptor's response to spike timing dependent plasticity (STDP) protocols, and thereby shape synaptic integration and information processing. This study investigates the role of GluN2 subunit differences on spine calcium concentration during several STDP protocols in a model of a striatal medium spiny projection neuron (MSPN). The multi-compartment, multi-channel model exhibits firing frequency, spike width, and latency to first spike similar to current clamp data from mouse dorsal striatum MSPN. We find that NMDAR-mediated calcium is dependent on GluN2 subunit type, action potential timing, duration of somatic depolarization, and number of action potentials. Furthermore, the model demonstrates that in MSPNs, GluN2A and GluN2B control which STDP intervals allow for substantial calcium elevation in spines. The model predicts that blocking GluN2B subunits would modulate the range of intervals that cause long term potentiation. We confirmed this prediction experimentally, demonstrating that blocking GluN2B in the striatum, narrows the range of STDP intervals that cause long term potentiation. This ability of the GluN2 subunit to modulate the shape of the STDP curve could underlie the role that GluN2 subunits play in learning and development.

## Author Summary

The striatum of the basal ganglia plays a key role in fluent motor control; pathology in this structure causes the motor symptoms of Parkinson's Disease and Huntington's Chorea. A putative cellular mechanism underlying learning of motor control is synaptic plasticity, which is an activity dependent change in synaptic strength. A known mediator of synaptic potentiation is calcium influx through the NMDA-type glutamate receptor. The NMDA receptor is sensitive to the timing of neuronal activity, allowing calcium influx only when glutamate release and a post-synaptic depolarization coincide temporally. The NMDA receptor is comprised of specific subunits that modify its sensitivity to neuronal activity and these subunits are altered in animal models of Parkinson's disease. Here we use a multi-compartmental model of a striatal neuron to investigate the effect of different NMDA subunits on calcium influx through the NMDA receptor. Simulations show that the subunit composition changes the temporal intervals that allow coincidence detection and strong calcium influx. Our experiments manipulating the dominate subunit in brain slices show that the subunit effect on calcium influx predicted by our computational model is mirrored by a change in the amount of potentiation that occurs in our experimental preparation.

## Introduction

The striatum is the main input structure of the basal ganglia, which is necessary for proper motor function and habit formation. The medium spiny projection neurons (MSPNs), which comprise ∼95% of striatal neurons, undergo changes in synaptic strength during the learning of a motor task [1]. This synaptic plasticity is thought to be the cellular basis of motor learning and habit formation, and it is disrupted in animal models of Parkinson's Disease [2] and Huntington's Disease [3].

One of the critical mechanisms for inducing synaptic plasticity in neurons is calcium elevation in the spine. The sources of calcium are quite diverse, and depend on brain region and direction of plasticity. In particular, LTD often requires release of calcium from internal stores [4] or voltage dependent calcium channels [4], [5]. In contrast, the source of spine calcium that contributes to long-term potentiation (LTP) is the NMDA receptor (NMDAR) in the hippocampus [6], cortex [7], and striatum [8].

Because NMDARs permit calcium influx in response to the coincidence of pre-synaptic glutamate release and post-synaptic depolarization, they are well situated to modulate spike timing dependent plasticity (STDP). In STDP protocols, an action potential (AP) is caused by depolarizing the soma of a neuron and is paired in time with a pre-synaptic stimulation. However, NMDARs differ in several properties that may be critical for timing-dependent synaptic plasticity. They contain various combinations of GluN1,2, and 3 subunits which can change their maximal conductance, current decay time, and sensitivity to magnesium block [9]. While the GluN1 splice variant has some control over the kinetic properties of the NMDAR, the four GluN2 subunits (A, B, C, and D) strongly control them when the GluN1 splice variant is kept the same [9]. The GluN2 subunit can thereby alter the calcium influx through the NMDAR. Because the specific differences between GluN2 subunits are the ones that would affect the NMDARs dependence on AP timing, and because calcium through the NMDAR plays an essential role in striatal timing-dependent long term potentiation (tLTP) [10]–[12] we hypothesized that changes in GluN2 subunit would modulate STDP in the striatum.

The MSPNs of the striatum contain both GluN2A and GluN2B subunits in abundance [13], and it has been suggested that GluN2D subunits may be present in low concentrations [14]. In animal models of Parkinson's disease, the NMDAR subunit composition is altered in the striatum [2] and subunit-specific NMDAR antagonists have been shown to alleviate Parkinson's like symptoms [15]. However, the intracellular consequences of such altered NMDAR subunit composition has not yet been made clear. In this study, we investigate the effects of altering NMDAR subunit composition on tLTP in the striatum.

Using a multi-compartmental model of a MSPN, we examine NMDAR-mediated calcium influx through receptors containing different GluN2 subunits and under different STDP conditions. We find that calcium elevation depends on which GluN2 subunit the NMDAR contains, the relative timing of the AP, the duration of somatic depolarization, and the number of consecutive APs. More significantly, model predictions about the effect of GluN2 subunit on the shape of the STDP curve are confirmed experimentally.

## Results

### Validation of model medium spiny projection neuron

**Figure 1:**
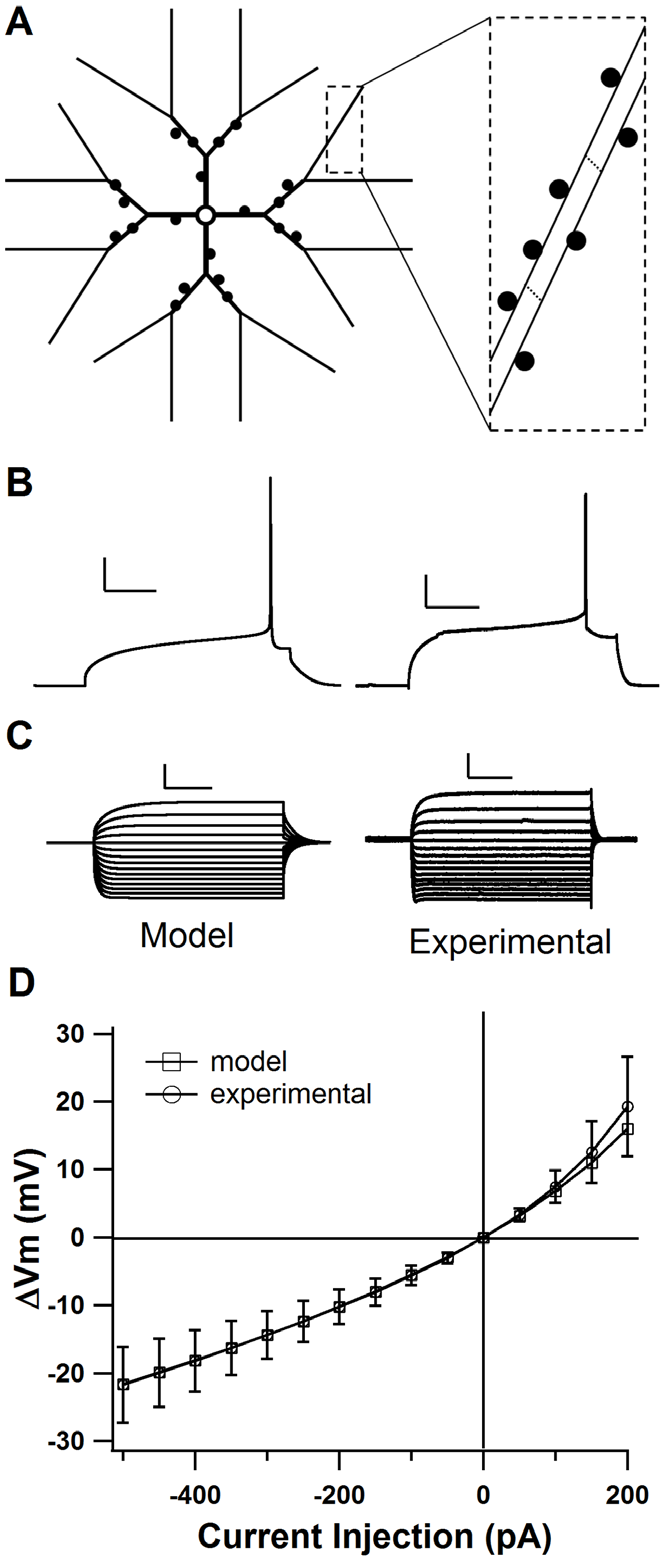
Model cell shows MSPN characteristics. **A**. Morphology of model MSPN (not to scale). Inset: tertiary dendrites have 11 segments each 36 µm in length. **B**. Comparison traces demonstrating latency to first AP in the model MSPN and an experimental whole-cell recording of a mouse MSPN. Both traces are a voltage response to current injection of 280 pA. Scale bars: 20 mV vertical, and 100 ms horizontal. **C**. Comparison current-voltage relationships (−500 pA to 200 pA) for the model MSPN and an experimental recording demonstrating inward rectification. Scale bars: 10 mV vertical, and 100 ms horizontal. **D**. current-voltage relationship for computational model compared with mean current-voltage relationship from 25 MSPNs of the mouse dorsal striatum. (See also Text S1: Supplemental Methods) Error bars ±SD.

To evaluate the role of the GluN2 subunit in STDP, we used a model of a dorsal striatal MSPN, which was modified from a nucleus accumbens neuron model [16], [17]. The model of the dorsal striatum MSPN had the same general morphology as the nucleus accumbens model (Figure 1A), with explicit spines that include synaptic glutamate receptors (AMPA and NMDA) and voltage dependent calcium channels. Kinetics and maximal conductances of voltage dependent channels (See Tables 1 and 2) were tuned to match the characteristics of neurons in the dorsal striatum, such as smallest current that evokes a single AP, long latency to first spike during somatic depolarization (Figure 1B), AP width of ∼1 ms, and distinct inward rectification (Figures 1C and see Text S1). Once the model was tuned to match these parameters, it also closely matched the average current-voltage relationship of 25 MSPNs from the mouse dorsal striatum undergoing a series of hyperpolarizing and depolarizing currents incrementing by 50 pA from −500 pA to 200 pA (Figure 1D and see Text S1). This model did not contain GABAergic synapses and represents experimental conditions in which GABAergic channels are blocked [10], [11], [18].

**Table 1:** Steady state and tau equations for ionic channels.

Channel Name	mh form	Steady State	tau	scale	α or β	Vhalf (mV)	Slope (mV)	Rate
NaF	m^3^	sigmoid	tab	1.2	-	−25	−9.2	1
	h	sigmoid	tab	1.2	-	−62	6	1
Kir	m	sigmoid	tab	0.5	-	−102	13	1
KaF	m^2^	α/(α+β)	1/(α+β)	1.5	α (sigmoid)	4	−17	1.5 ms^−1^
					β (sigmoid)	10	9	0.6 ms^−1^
	h	α/(α+β)	1/(α+β)	0.67	α (sigmoid)	−121	22	105 s^−1^
					β (sigmoid)	−55	−11	65 s^−1^
KaS	m^2^	α/(α+β)	1/(α+β)	2.5	α (sigmoid)	50	−20	250 s^−1^
					β (sigmoid)	−90	35	50 s^−1^
	h	α/(α+β)	1/(α+β)	2.5	α (sigmoid)	−95	16	2.5 s^−1^
					β (sigmoid)	50	−70	2 s^−1^
Kdr	m	1/(1+a)	0.05*b/(1−a) s	0.5	a (exp)	−13	9.09	-
					b (exp)	−13	12.5	-
BK	m	α/(α+β)	1/(α+β)	1	α (BK_eqn1)	D = −0.84, K = 0.003, B = 480 s^−1^		
					β (BK_eqn2)	D = −1, K = 0.009, B = 280 s^−1^		
SK	m	SK_eqn	4e-3	1	-	*EC50* = 0.57 µM		

Voltage sensitive calcium channels are not listed because they use the same parameters and equations as Wolf et al. [16], but using m instead of m^x^ for CaL1.2, CaN, CaR, and CaT. V = voltage, scale = value by which time constants were scaled during tuning, [Ca] = calcium concentration, EC_50_ = half activation for calcium dependence, F = Faraday constant, R = Ideal Gas constant. Equations: sigmoid = rate/(1+(exp ((V−vhalf)/slope))), exp = exp((V−vhalf)/slope), SK_eqn = (([Ca]/*EC50*)∧5.4)/(1+([Ca]/*EC50*)∧5.4), BK_eqn1 = (B*[Ca])/([Ca]+K*(exp((2VDF)/(RT)), BK_eqn2 = B/(1+[Ca]/(K*(exp((2VDF)/(RT)).

**Table 2:** Maximal conductance and permeability for ionic channels.

Gbar (S/M^2^)	soma	prox dend	mid dend	dist dend	spine
**NaF**	90,000	2,730	2,730	975	0
**KaF**	765.24	765.24	168.21	112.14	0
**KaS**	360.1	38.93	38.93	38.93	0
**Kir**	8	8	8	8	0
**KDR**	6.04	6.04	6.04	6.04	0
**SK**	2	2	2	2	0
**BK**	10	10	10	10	0
**Pbar**					
**CaL1.3**	1.59E-07	1.59E-07	1.59E-07	1.59E-07	4.25E-07
**CaT**	8.81E-09	8.81E-09	8.81E-09	8.81E-09	2.35E-08
**CaR**	9.75E-07	9.75E-07	9.75E-07	9.75E-07	1.30E-06
**CaN**	3.75E-07	3.75E-07	3.75E-07	3.75E-07	0
**CaL1.2**	1.26E-07	1.26E-07	1.26E-07	1.26E-07	3.35E-07

Gbar = maximal conductance S/M^2^ = Siemans per meter squared; Pbar = maximal calcium permeability. Prox dend = proximal dendrites; mid dend = middle dendrites; dist dend = distal dendrites.

### STDP protocol type modulates calcium influx through NMDAR

Because NMDAR channel opening requires both glutamate to activate the channel and membrane depolarization to relieve the voltage-dependent magnesium block, the occurrence of a somatic AP close in time to glutamatergic stimulation can drastically increase the calcium elevation due to the NMDAR. Previous studies have shown that different somatic depolarization patterns back-propagate into the dendrites with varying strength [19]–[21]. Consequently, STDP protocols that have been used on striatal cells vary in AP number and length of somatic depolarization.

**Figure 2:**
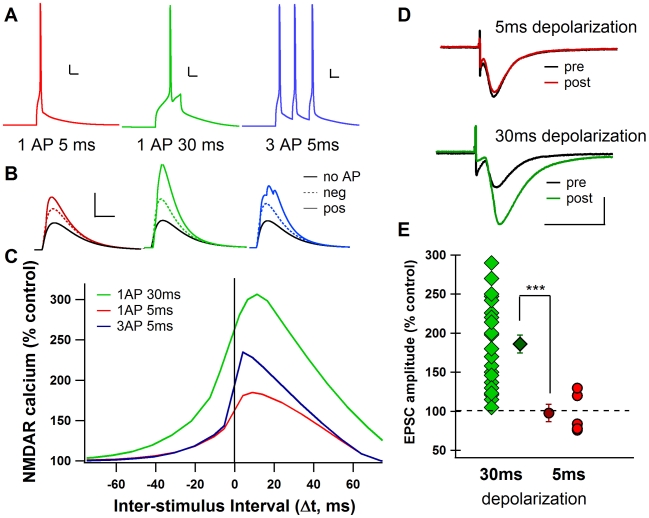
STDP protocol type changes calcium influx through NMDAR. **A**. Model traces showing each STDP protocol. Scale bars: 10 mV vertical, 10 ms horizontal. **B**. NMDAR-mediated calcium during positive (solid color line) and negative (dotted color line) Δt compared to calcium through NMDA during synaptic stimulation only (No AP, solid black line). Scale bars: 2 µM [Ca^2+^] vertical, 50 ms horizontal. **C**. Model calcium curves showing change in peak NMDAR calcium as a percentage of control (synapse stimulation with no AP). Spines stimulated are 40 µm from the soma. **D**. Example single experiments showing tLTP in response to 30 ms depolarization STDP, but not to 5 ms depolarization STDP. pre = average of baseline EPSCs prior to STDP; post = average of EPSCs around 60 minutes after STDP. Scale bars: 100 pA vertical, 20 ms horizontal. **E**. All experimental data points approximately 60 minutes after STDP protocol application as % of baseline. 5 ms depolarization STDP failed to induced significant tLTP when compared to 30 ms depolarization STDP. (*** = p<0.0001).

To investigate the influence that different protocols have on the NMDAR-mediated calcium elevation in the spine, simulations were performed for each STDP protocol that has been used in the striatum: a 30 ms depolarization to 1 AP [10], [22], a 5 ms depolarization to 1 AP [11], and an AP triplet at a frequency of 50 Hz [11], [18] (Figure 2A). Each simulation involved the stimulation of two adjacent spines on the secondary dendrite of the model cell and a somatic depolarization to AP initiation, with the two events separated by a specific temporal interval (Δt). For each STDP protocol, the peak calcium elevation due to the NMDAR in a single spine was recorded for both positive and negative Δt and was compared to the peak NMDAR-mediated calcium elevation due to control stimulation with no somatic AP (Figure 2B). When repeated over a range of Δt, simulations reveal that a 30 ms depolarization to 1 AP significantly increased the NMDAR-mediated calcium in the spine when it occurred +2 to +20 ms after pre-synaptic stimulation (∼250% increase), while a 5 ms depolarization to AP caused much reduced elevations in NMDAR-mediated calcium at these intervals (∼175% increase). The NMDAR-mediated calcium elevation observed with negative Δt was reduced compared to positive Δt for all three protocols, consistent with the experimental observation that in the presence of GABA inhibitors, negative Δt does not produce tLTP [10]. Because in reality, the AP does not always occur at the exact same time within the depolarization, STDP simulations were repeated with added random synaptic input to cause noise and jitter to the timing of the AP within the 30 ms depolarization. Simulations were repeated 6 times using different input frequencies and random seed values. The averages of these simulations overlay the control conditions, demonstrating that this model and protocol are robust to noise (Figure S1).

Because calcium through the NMDAR is essential for tLTP [10], [12], the model predicts that the 30 ms depolarization protocol, which causes a strong increase in NMDAR-mediated calcium, will more readily induce tLTP than the 5 ms depolarization protocol which causes a weak increase in NMDAR-mediated calcium. This model prediction was confirmed experimentally with whole-cell patch-clamp recordings in rat striatal slices. STDP experiments show that using Δt of +5 to +30 ms, robust tLTP is induced with the 30 ms depolarization protocol, but tLTP is not induced with the 5 ms depolarization protocol (Figures 2D &E). Indeed, with the 30 ms depolarization protocol, the mean EPSC amplitude value, recorded 60 minutes after the induction protocol, was 186.0±11.6%, (Δt = +18.6±1.5 ms, n = 22), a value significantly different (p<0.01) from baseline (Figure 2E). However, 5 ms postsynaptic suprathreshold depolarizations paired with presynaptic stimulations (Δt = +10.3±0.4 ms, n = 5) were not able to induce significant plasticity. The mean EPSC amplitude recorded 60 minutes after the induction protocol (97.5±11.0%) did not significantly differ from baseline (Figure 2E).

### GluN2 subunit composition modulates calcium elevation due to NMDAR

The type of GluN2 subunit (A, B, C, and D) strongly determines the maximal conductance, decay time, and sensitivity to magnesium block for the NMDAR [9]. Therefore, differences in GluN2 subunit are predicted to modulate synaptic plasticity by controlling the calcium influx through the NMDAR. To evaluate this hypothesis, simulations were repeated using the 30 ms depolarization STDP protocol that caused tLTP for four types of NMDAR each representing a receptor containing two GluN1 subunits and two GluN2 subunits of the same type (either A, B, C, or D). The current decay time, maximal conductance, and magnesium block parameters of these NMDARs were adjusted to reflect experimental measurements [23], [24] (Table 3, Figures S2A & B).

**Table 3:** NMDA subunit parameters.

	GluN2A	GluN2B	GluN2C	GluN2D
**Gmax (S)**	0.94e-9	0.94e-9	0.325e-9	0.119e-9
**Tau decay (s)**	25e-3	150e-3	125e-3	850e-3
**Mg^2+^ block**	3.57	3.57	25	40

S = Siemens, s = seconds. “Mg^2+^ block” is parameter A used in equation 2 (See methods and also Figure S2).

**Figure 3:**
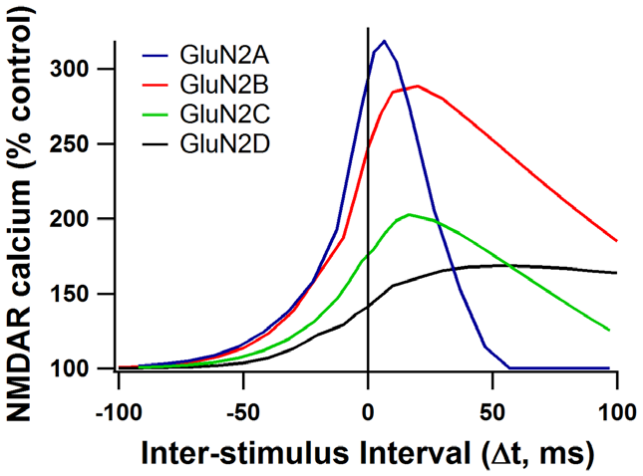
Calcium curves for different GluN2 subunit containing NMDARs. Dependence of NMDAR-mediated calcium on AP timing is different for each GluN2 subunit. Normalized peak NMDAR calcium is plotted for each AP timing interval (Δt) using the 30 ms STDP protocol. Spines stimulated are 40 µm from the soma.

Calcium elevation due to NMDAR was evaluated for each GluN2 subunit condition over all Δt (from −100 to +100 ms). The resulting calcium curves (Figure 3) show clear differences in shape depending on GluN2 subunit. GluN2A and GluN2B conditions show the highest normalized calcium peaks, reaching greater than 250% of control, while GluN2C demonstrates a much reduced increase in calcium due to AP timing, just reaching 200% at the narrowest intervals. Similarly, GluN2D exhibits reduced calcium peaks and shows very little change in calcium concentration based on the AP timing (Figure 3). These simulations suggest that tLTP would be readily induced in GluN2A and GluN2B-containing synapses, but would not be as easily induced in GluN2C and GluN2D-containing synapses.

Although GluN2A and GluN2B-containing receptors both reach calcium peaks greater than 250%, the shapes of the calcium curves are strikingly different. Because of the faster decay time of GluN2A, the range of Δt that causes strong calcium elevation is much narrower than for the more slowly decaying GluN2B-containing receptors. Calcium elevation due to influx through GluN2A only reaches levels >250% for narrow Δt. However, the GluN2B-containing receptors maintain elevations in calcium >250% even at wide Δt (Figure 3).

**Figure 4:**
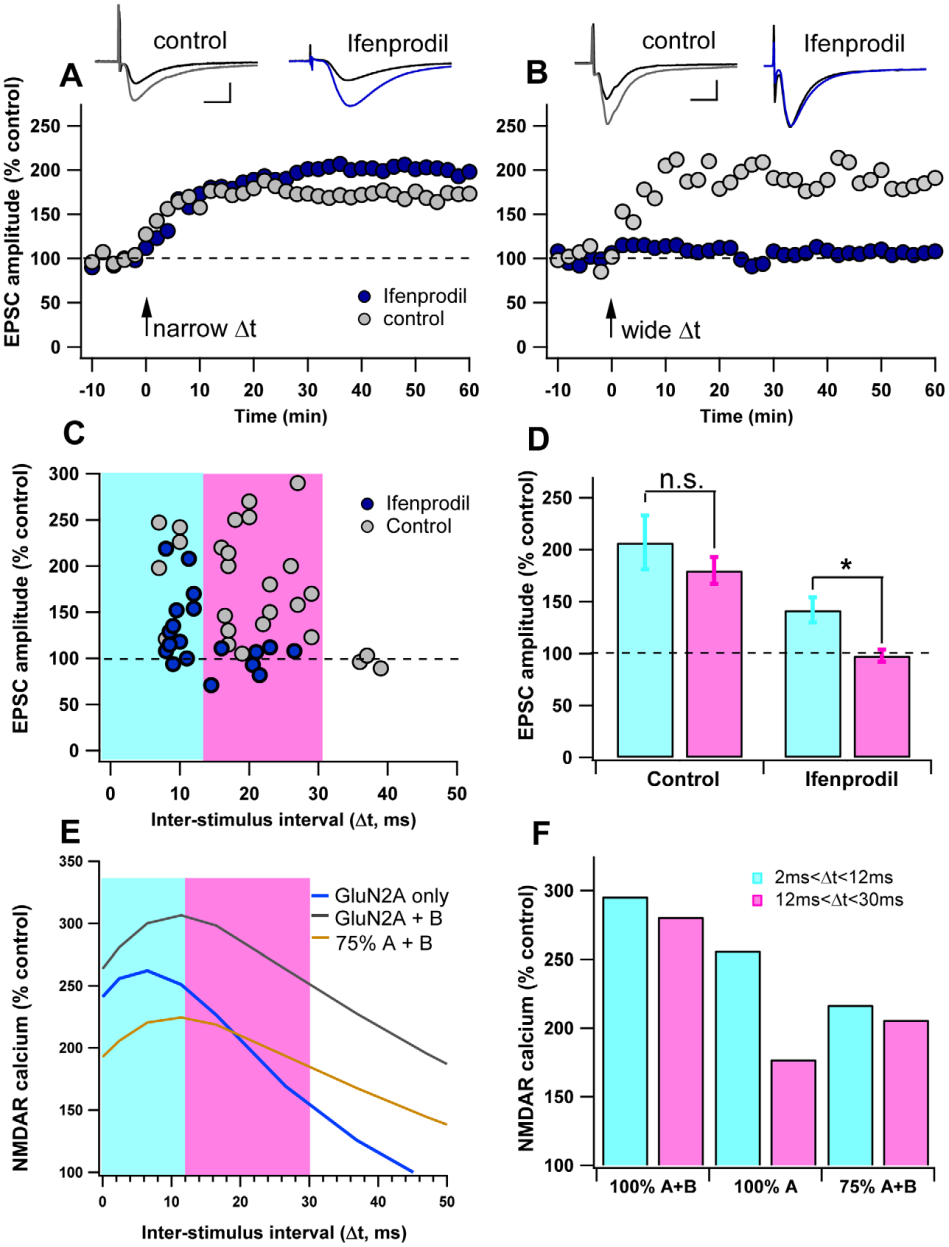
GluN2B broadens the STDP curve. **A–B**. Inhibition of GluN2B restricts the time window of tLTP induction. Representative experiments illustrate the time course of synaptic efficacy changes induced by pre-post pairings in control aCSF and in the presence of ifenprodil (10 µM). Insets: averaged EPSCs before (black) or after (grey: control; blue: ifenprodil treatment) STDP. Scale bars 100 pA vertical, 10 ms horizontal. **A.** Example experiments showing tLTP induced by narrow AP timing intervals (0<Δt<12 ms) in control and ifenprodil conditions. **B.** Example experiments showing tLTP is induced by wide AP timing intervals (12<Δt<30 ms) in control conditions, but not induced when GluN2B containing NMDARs were blocked by Ifenprodil. Black arrow indicates the STDP protocol induction. **C.** Summary of Experimental data: Spike-timing dependent changes in synaptic efficacy estimated 60 minutes after STDP induction in control and Ifenprodil conditions. Blue shading highlights Δt shorter than 12 ms; pink shading highlights Δt between 12 ms and 30 ms. **D.** Bar graph of long-term synaptic efficacy changes shows that the GluN2B inhibition affects the range of Δt that permits tLTP induction. (* = p<0.05, n.s. = not significant) **E.** Peak NMDAR calcium curves from the model MSPN for positive Δt. All curves are normalized to the GluN2A+B no AP condition. **F.** Bar graph showing average NMDA calcium elevation for narrow Δt (+2 ms to +12 ms) and wide Δt. (+13 ms to +30 ms) for each NMDAR condition (GluN2A+B, GluN2A alone, and 75% GluN2A+B).

Because the predicted intervals that allow significant amounts of calcium through NMDARs differ between GluN2A and GluN2B and because striatal MSPNs contain both GluN2B and GluN2A subunits [25], the model predicts that blocking the GluN2B subunit in an MSPN would narrow the range of Δt that produces tLTP. To test this prediction, STDP experiments were conducted in either normal aCSF, or in the presence of 10 µM Ifenprodil, a potent and selective GluN2B antagonist. In the control condition, Δt between +5 ms and +30 ms elicits tLTP (186.0±11.6%, n = 22) (Figure 4A–D). This Δt range includes both narrow timing intervals (5 ms<Δt<12 ms), and wide timing intervals (12 ms<Δt<30 ms). Under control aCSF conditions, the tLTP induced using narrow Δt (207±26%, n = 5) does not significantly differ from the tLTP induced using wide Δt (179.9±13.4%, n = 17) (P = 0.72). In contrast, when GluN2B-containing NMDARs are blocked with Ifenprodil, tLTP at intervals wider than +12 ms is abolished (97.7±6%, n = 7), while tLTP between +5 ms and +12 ms was maintained (142±12%, n = 12). This difference between wide and narrow timing intervals is significant for the Ifenprodil condition (p<0.05; Figure 4D). These results demonstrate that GluN2 subunits not only control *whether* potentiation occurs, as previous studies have shown [26]–[28], but also that they *hone* plasticity, making it sensitive to wider or narrower time intervals between pre-synaptic neurotransmitter release and post-synaptic firing.

To reflect these experiments in the model cell, control aCSF simulations were run using a combined GluN2A and B decay time constant (25% GluN2B, 75% GluN2A [25]), and compared to the GluN2A only condition. For both cases the calcium response was normalized by the control aCSF no AP condition.

The calcium curves generated by the model MSPN reflect the experimental STDP curves. When GluN2A and GluN2B are present, calcium elevation due to NMDAR stays above 250% of control for both narrow (2 ms<Δt<12 ms) and wide (12 ms<Δt<30 ms) Δt, whereas when GluN2A is present alone, calcium elevation due to NMDAR drops below 250% of control for wide timing intervals (Figures 4E&F). Interestingly, when GluN2A and GluN2B are present, the interval at which NMDAR-mediated calcium drops below 250% in the model (+40 ms Δt) is the same interval that no longer gives tLTP in the experiments (Figures 4C &E).

During the narrow timing intervals (5 ms<Δt<12 ms), Ifenprodil significantly reduced the amount of tLTP induced for the experiments, and reduced the NMDAR mediated calcium in the model simulations. This result is not surprising considering that the synapses in the Ifenprodil condition have fewer total NMDARs available than those in the control condition. To test whether a general 75% reduction in NMDAR conductance would produce the same calcium effects as the GluN2A only condition, simulations were run using 75% of the NMDAR conductance and the combined GluN2A+B kinetics (Figures 4E&F). This general reduction reduced the effect of AP pairing, but did not reproduce the timing difference seen with the GluN2A only case. Our model predicts that if NMDAR conductance is reduced by 75%, LTP would be reduced for both narrow and wide Δt.

### Spines on distal dendrites show reduced sensitivity to AP timing

**Figure 5:**
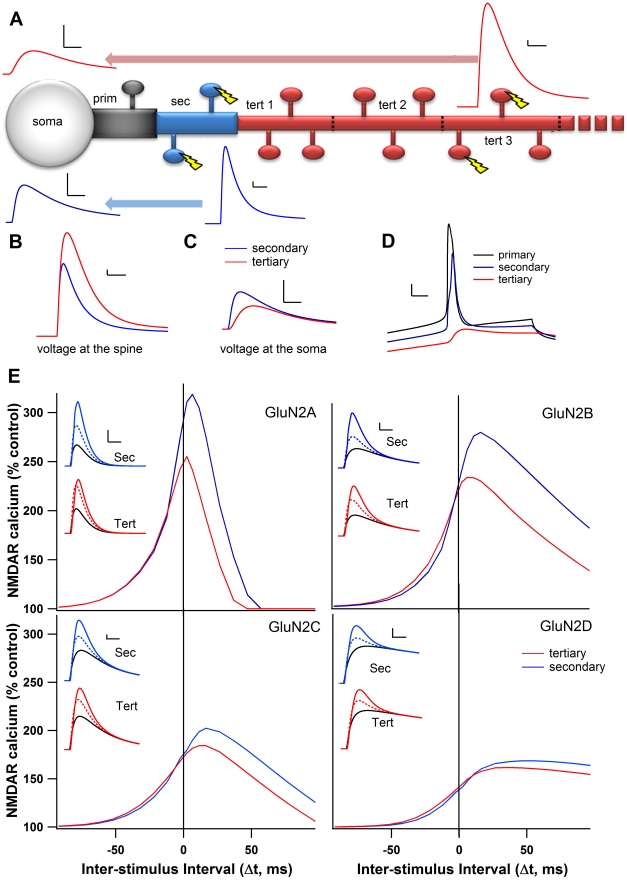
Distance from soma reduces dependence on AP timing. **A**. Illustration of dendritic branch, showing the decay of the EPSP traveling from either the tertiary (red) or secondary (blue) dendritic spines. In all panels “tertiary” refers to the third segment of the tertiary dendrite (tert 3). Scale bars 0.5 mV vertical, 10 ms horizontal. **B**. Overlay of tertiary and secondary EPSPs as seen at the spine. Scale bars: 0.5 mV vertical, 10 ms horizontal. **C**. Overlay of tertiary and secondary EPSPs for the same stimulations in **B** as seen at the soma. Scale bars: 0.5 mV, 10 ms **D**. Spine depolarizations resulting from the back-propagating AP for the primary, secondary, and tertiary dendrites. Scale bars: 10 mV vertical, 5 ms horizontal. **E**. Peak NMDAR calcium curves for each GluN2 subunit on the tertiary dendrite and the secondary dendrite. Insets: NMDAR-mediated calcium traces for secondary (top) and tertiary (bottom) dendrites for positive (Δt = +11 ms, solid color), negative (Δt = −12 ms, dotted color) and no AP control conditions (solid black). Scale bars 1 µM, vertical, 10 ms horizontal.

Previous studies in other neuronal types have shown that distance from the soma alters the strength [29] and direction of STDP [30]. Because the AP decays as it back-propagates in MSPNs [31], we hypothesized that the timing dependence seen in secondary dendrites of these neurons would shift with distance from the soma. To investigate these interactions, we evaluated the peak calcium elevation during the 30 ms depolarization STDP protocol while stimulating spines located on the third segment of the tertiary dendrite, 150 µm away from the soma, compared to the secondary dendrites at 40 µm. Because a smaller branch diameter increases the input resistance at the tertiary dendrite, stimulating two adjacent tertiary spines resulted in a larger post-synaptic potential (PSP) than stimulating the two adjacent secondary spines, when seen at the spine head (Figures 5A & B). However, the greater electrotonic distance for the tertiary stimulation causes the same stimulation to be smaller when seen at the soma (Figures 5A&C). As predicted by cable theory [32], the depolarization seen in the spine due to the back propagating AP is smaller in the tertiary dendrites than in the secondary dendrites (Figure 5D).

A reduced sensitivity to AP timing for positive Δt is observed under every GluN2 subunit condition (Figure 5E), due to both the decrement in back-propagating AP, which decreases the amplitude of the NMDAR-mediated calcium elevation, and the larger PSP, which decreases the need for depolarization by the AP. In contrast, virtually no change in calcium elevation or timing dependence appears for negative Δt (Figure 5E). The most drastic decreases in NMDAR-mediated calcium elevation occurs in the subunit conditions most sensitive to the magnesium block, i.e. GluN2A and GluN2B, while the GluN2C and GluN2D subunit conditions showed a smaller decrease in NMDAR-mediated calcium elevation due to distance. Because there is no change for negative Δt and a decrease in peak calcium for positive Δt, the difference between positive and negative Δt is strongly reduced when synapses are on the tertiary dendrite. For example, when GluN2A is stimulated on the tertiary spine, positive Δt causes nearly the same elevation in calcium as negative Δt (Figure 5E inset). Under these distal dendrite conditions, positive Δt is unlikely to produce tLTP, and the dependence on AP timing is drastically reduced.

### Differences between thalamo-striatal and cortico-striatal synapses

The MSPNs of the striatum receive inputs from almost all areas of the cortex [33] and from the thalamus [34]. Using a recently developed slice preparation which preserves both cortico-striatal and cortico-thalamic fibers [35], Ding et al. [25] and Smeal et al. [36] have characterized the two major glutamatergic inputs to the striatum in mouse and rat respectively. Ding et al. found that thalamo-striatal synapses had a lower NMDAR/AMPAR ratio than cortico-striatal synapses in the mouse, while Smeal et al. found the opposite, that the NMDAR/AMPAR ratio was higher for thalamo-striatal synapses in the rat. Measuring the decay time constant, Smeal et al. found that thalamo-striatal synapses had lower GluN2B/NMDAR ratios than cortico-striatal synapses, while using bath application of Ifenprodil Ding et al. found the opposite, that thalamo-striatal synapses had a higher GluN2B/NMDAR ratio. Smeal et al. also found that the thalamo-striatal synapses were electrotonically more distant from the soma than the cortico-striatal synapses, Ding et al. did not measure this (Table 4). To investigate the implications of differences in the NMDAR/AMPAR ratio, the GluN2B/NMDAR ratio, and the electrotonic distance from the soma, we modeled synapses with specific cortico-striatal characteristics and specific thalamo-striatal characteristics. Because the two studies report contrasting results [25], [36], we ran separate simulations using each set of data.

**Table 4:** Cortico-striatal and thalamo-striatal parameters.

	NMDAR/AMPAR			GluN2B/NMDAR			Distance from soma
**Smeal et al., 2008**	Ctx	1.52	↓	Ctx	τ = 116.5 ms	↑	Ctx more proximal
	Th	2.6	↑	Th	τ = 96.5 ms	↓	Th more distal
**Ding et al., 2008**	Ctx	2.75	↑	Ctx	0.25	↓	–*Not Addressed*–
	Th	2.04	↓	Th	0.42	↑	–*Not Addressed*–

Ctx = cortico-striatal synapses; Th = thalamo-striatal synapses. τ = decay time constant for NMDA current. Arrows represent a relative increase or decrease in NMDA/AMPA ratio or GluN2B/NMDA ratio between cortico-striatal and thalamo-striatal synapses for each study.

**Figure 6:**
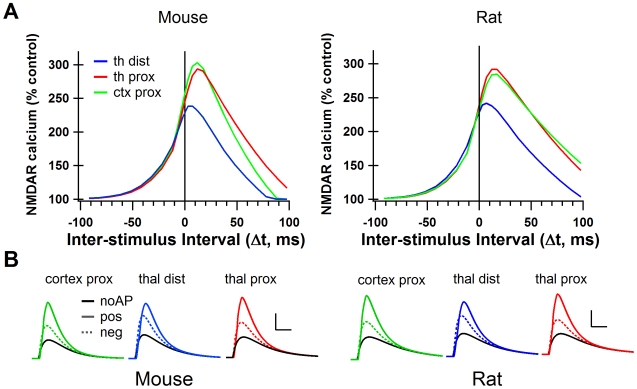
The cortico-striatal and thalamo-striatal synapses onto MSPNs have different synaptic characteristics. **A**. Peak NMDAR calcium curves from simulations using cortico-striatal and thalamo-striatal parameters from mouse striatum (Ding et al., 2008) and from rat striatum (Smeal et al., 2008). Cortico-striatal curve for mouse is the same as the green trace in figure 2C. **B**. NMDAR-mediated calcium for positive (Δt = +11 ms, solid color), negative (Δt = −12 ms, dotted color) and no AP control conditions (solid black) under cortico-striatal and thalamo-striatal conditions in mouse and rat. Scale bars: 2 µM [Ca^2+^] vertical, 50 ms horizontal. thal dist = thalamo-striatal synapses stimulated on tertiary dendritic spines, thal prox = thalamo-striatal synapses stimulated on secondary dendritic spines. cortex prox = cortico-striatal synapses stimulated on secondary dendritic spines.

We found that, based on different electrotonic properties, thalamo-striatal and cortico-striatal synapses differentially affect the NMDAR mediated calcium elevations for positive Δt, but not negative Δt. The distal thalamo-striatal synapses had lower peak calcium, and a weaker dependence on AP timing than did the cortico-striatal synapses for both mouse and rat (Figure 6A). This result suggests that NMDAR-dependent tLTP would be more readily induced in cortico-striatal synapses than in thalamo-striatal. However, this result assumes that thalamo-striatal synapses are more distal than the cortico-striatal as has been experimentally suggested [36]. To distinguish the effect of distance from the effect of NMDAR/AMPAR and GluN2B/NMDAR ratios, simulations were repeated with thalamo-striatal synapses in the same location (secondary dendrite) as the cortico-striatal synapses. When the synapses were located at the same distance from the soma, the effect of Δt on peak calcium concentration did not differ strongly between thalamo-striatal and cortico-striatal synapses (Figure 6A). Therefore, the lower calcium peaks in the thalamo-striatal simulations are entirely due to its location on the tertiary dendrite. Interestingly, this result was independent of which dataset (Ding et al. or Smeal et al.) was used.

In conclusion, we predict that the cortico-striatal and thalamo-striatal synapses onto an MSPN will respond similarly to STDP protocols if they are located a similar distance away from the soma. However, if as Smeal et al. [36] have shown, the thalamo-striatal synapses are more distal, then we predict it will be more difficult to induce NMDAR dependent tLTP in those synapses than in cortico-striatal synapses.

## Discussion

We developed a multi-compartment, multi-channel model of a dorso-striatal MSPN to investigate the relationship between NMDAR mediated calcium and tLTP. The NMDAR is critical for learning and STDP because it admits calcium in response to the coincidence of pre-synaptic activity and post-synaptic depolarization. It is clear that other mechanisms besides calcium are important for long term synaptic plasticity [37]. While there are many mechanisms to increase calcium in the cell, such as voltage sensitive calcium channels, calcium permeable AMPA receptors, and calcium release from internal stores, these mechanisms do not all serve the same function [38] and not all sources of calcium contribute to tLTP [4], [10]. NMDAR-mediated calcium has consistently been shown to be essential for tLTP in the striatum [10], [11], [18], while it is not necessary for tLTD [5], [10], [12]. Therefore, the NMDAR-mediated calcium elevations recorded from this model are used to predict tLTP, but are not relevant to the development of tLTD.

We found that a long depolarization to AP resulted in greater calcium increases (in the model) and more tLTP (experimentally) than a short depolarization to AP. In cortex and hippocampus, a short depolarization often is sufficient to induce tLTP [4], [39]. However, MSPNs usually receive inputs over a longer time-course which generate an upstate and AP [40], [41]. Thus, a long depolarization to AP mimics a more natural time course for these cells. One study was able to induce tLTP in the striatum with a short depolarization to AP, and found the same degree of tLTP with the three AP protocol [11]. However, these experiments were conducted in the presence of glycine, a NMDA co-agonist, which could have increased NMDAR sensitivity [42].

### Distance from the soma and STDP

In the model, NMDAR-mediated calcium elevations show strong sensitivity to AP timing in proximal dendrites, but this sensitivity as well as the maximum change in calcium is diminished at distal synapses, independent of STDP protocols and GluN2 subunit. Given the diminished effect of AP timing in tertiary dendrites, LTP at distal synapses might be achieved through entirely different plasticity mechanisms not requiring a somatic AP. A recent study suggests that a limited number of inputs on distal dendritic branches can induce upstates in MSPNs due to the high input resistance of the branch and non-linearity of the NMDA receptor [43]. Thus, the conjunction of many synaptic inputs may be more important than somatic depolarization for distal synaptic plasticity. However, further work is needed to investigate whether this upstate induction can induce LTP at synapses distal to the soma.

We have used the recently characterized thalamo-striatal and cortico-striatal inputs to MSPNs to model synapses of each type. Simulations show that distance from the soma was the only factor that created a differential response to STDP protocols between these two types of synapse. Thus, our results predict that any disparity in plasticity expression between these synapses will not be NMDAR related, and will come from other neuromodulatory mechanisms, such as differences in pre-synaptic endocannabinoid receptor expression [44].

### GluN2 subunits and their effects on synaptic plasticity

Our model shows that the time course and voltage dependence of GluN2 subunit can influence the way synapses respond to STDP protocols. GluN2A and GluN2B produce a stronger sensitivity to AP timing than GluN2C and GluN2D. Although the striatum contains mostly GluN2A and GluN2B subunits, our findings predict that neuron types with predominantly GluN2C or GluN2D will not exhibit NMDAR dependent tLTP. In addition, GluN2A, because of its fast decay time, results in a narrowing of the STDP curve (compared to GluN2B) by decreasing the Δt that permits sufficient calcium influx. While previous studies have focused on *whether* a particular GluN2 subunit is necessary for plasticity [26]–[28], we have shown that the relationship between GluN2 subunit and plasticity is more complex than simply allowing or preventing LTP.

In addition to the basic subunit characteristics modeled here, there are other, less well understood parameters that differ between the four GluN2 subunits. For example, differing amino acid patterns in the c-terminal tail allow differential phosphorylation by kinases and differential binding to scaffolding proteins [45]. Some of these differences are known to change the calcium permeability of the channel [46], and could further influence the receptor's response to STDP. Similarly, the intracellular location of the GluN2 subunits is not well known in the striatum. A few studies have looked at the synaptic versus extrasynaptic location of GluN2A and GluN2B [47], [48] in the striatum, but the techniques used were comparative rather than quantitative; thus we cannot be certain that we are not stimulating extrasynaptic NMDARs, but the low frequency stimulation (always 1 Hz or lower) used here is unlikely to cause significant glutamate spillover [49]. It is possible that NMDA triheteromers (containing both GluN2A and B) are present in the striatum ([50], but see [25]). Our experimental protocol does not distinguish between di or triheteromers and therefore we cannot specifically determine whether the effect is due to Ifenprodil's full blockade of GluN2B diheteromers or its weak blockade of GluN2A/B triheteromers [51].

The balance between GluN2A and GluN2B, and thus the shape of the STDP curve may be modulated dynamically. Studies in the hippocampus have demonstrated a widening of the STDP curve in response to dopamine D1 receptor stimulation [52], and β-adrenergic receptor stimulation [53]. Interestingly, both D1 and β-adrenergic receptors activate the cAMP-dependent protein kinase (PKA) which is essential for striatal LTP [54]. PKA phosphorylation is known to increase the calcium permeability of NMDARs and in particular, those containing GluN2B subunits [46]. Therefore, PKA activation may widen the STDP curve by increasing the calcium permeability of GluN2B-containing NMDARs.

### Implications for learning, behavior, and pathology

The role for NMDAR subunits in neurological disorders has recently been suggested. Studies conducted in rodent models of Parkinson's Disease have shown that dopamine depletion results in the reconfiguration of the NMDARs, specifically in a reduction of GluN2B subunits [47]. Other studies have found that the administration of GluN2B antagonists reduces dyskinesia in animal models of Parkinson's Disease while GluN2A antagonists may increase it [55]. Similarly, GluN2B containing receptors are selectively potentiated by mutant huntingtin [56], suggesting abnormal GluN2B subunit activity in Huntington's Disease. An imbalance of GluN2B containing NMDARs may allow non-specific potentiation that could lead to excessive and uncontrolled movement. Our results contribute to the emerging picture of GluN2 subunit antagonists as treatments for neurological disorders of the striatum by elucidating a possible mechanism for GluN2 subunit alterations to alter striatal plasticity and therefore motor behavior.

Our findings suggest a novel role for the GluN2A NMDAR subunit in striatal synaptic plasticity. Instead of allowing or preventing LTP, this subunit *hones* plasticity, narrowing the STDP curve and allowing for the fine-tuning of neuronal pathway strength. Previous work has shown that the medial striatum undergoes plastic changes during the early, more coarse, phase of skill learning, while the lateral striatum undergoes plasticity during the late, fine-tuning, phase of skill learning [1]. Interestingly, the lateral striatum contains a higher ratio of GluN2A to GluN2B subunits than the medial striatum [13]. A high density of the GluN2A subunit may functionally underlie the fine-tuning phase of skill learning, allowing potentiation of only the most closely-timed connections. While the higher ratio of GluN2B subunits in the medial striatum would be useful for less specific, but possibly faster acquisition of a skill. This role for GluN2A may also underlie the experience-dependent developmental shift from GluN2B to GluN2A in the visual cortex [57], and may be responsible for the increase in spatial learning ability that coincides with the developmental switch from GluN2B to GluN2A at hippocampal synapses [58].

## Methods

### Ethics statement

All plasticity experiments were performed in accordance with local animal welfare committee (Center for Interdisciplinary Research in Biology and College de France) and EU guidelines (directive 86/609/EEC). For electrophysiology used to tune the computational model, animal handling and procedures were approved by the George Mason University IACUC committee (Text S1). Every precaution was taken to minimize stress and the number of animals used in each series of experiments.

### Electrophysiology

Animals, OFA rats (Charles River, L'Arbresle, France) (postnatal days 17–25) were sacrificed by decapitation and brains were immediately removed. Patch-clamp recordings of MSPNs were performed in horizontal brain slices (330 µm) from OFA rats. These horizontal slices included the somatosensory cortical area and the corresponding cortico-striatal projection field [22] and were prepared with a vibrating blade microtome (VT1000S and VT1200S, Leica Micosystems, Nussloch, Germany). Patch-clamp whole-cell recordings were performed in the somatosensory area of the dorsal striatum and made as previously described [10], [22]. Briefly, borosilicate glass pipettes of 5–7 MΩ resistance contained (mM): 105 K-gluconate, 30 KCl, 10 HEPES, 10 phosphocreatine, 4 ATP-Mg, 0.3 GTP-Na, 0.3 EGTA (adjusted to pH 7.35 with KOH). The composition of the extracellular solution was (mM): 125 NaCl, 2.5 KCl, 25 glucose, 25 NaHCO_3_, 1.25 NaH_2_PO_4_, 2 CaCl_2_, 1 MgCl_2_, 10 µM pyruvic acid bubbled with 95% O_2_ and 5% CO_2_. Picrotoxin (50 µM) (Sigma, Saint Quentin, France) was dissolved in ethanol and then added in the external solution for a final ethanol concentration of 0.01%. All recordings were performed at 34°C using a temperature control system (Bioptechs ΔTC3, Butler, PA, USA and Bath-controller, Luigs&Neumann, Ratingen, Germany) and slices were continuously superfused at 2–3 ml/min with the extracellular solution. Individual neurons were identified using infrared-differential interference contrast microscopy with CCD camera (Hamamatsu C2400-07; Hamamatsu, Japan). Signals were amplified using EPC10-2 amplifiers (HEKA Elektronik, Lambrecht, Germany). Current-clamp recordings were filtered at 2.5 kHz and sampled at 5 kHz, and voltage-clamp recordings were filtered at 5 kHz and sampled at 10 kHz using the program Patchmaster v2x32 (HEKA Elektronik). The series resistance was compensated at 75–80%.

### Spike timing-dependent plasticity induction protocols

Electrical stimulation of the cerebral cortex was performed with a bipolar electrode (Phymep, Paris, France) placed in the layer 5 of the somatosensory cortex [22]. Electrical stimulations were monophasic at constant current (Stimulator WPI, Stevenage, UK or ISO-Flex stimulator controlled by a Master-8, A. M. P. I., Jerusalem, Israel). Currents were adjusted in order to evoke striatal excitatory postsynaptic currents (EPSCs) ranging in amplitude from 50 to 200 pA. Repetitive control stimuli were applied at 0.1 Hz, a frequency for which neither short- nor long-term synaptic efficacy changes in EPSC amplitudes were induced [22].

STDP protocols consisted in pairings of pre- and post-synaptic stimulations with the two events separated by a specific temporal interval (Δt) repeated 100 times at 1 Hz. Pre-synaptic stimulations correspond to cortical stimulations and the post-synaptic stimulation to an AP evoked by a direct application of a depolarizing current step (5 or 30 ms duration) in the MSPN. Neurons were recorded for 10 minutes during baseline and for at least 60 minutes after the cellular conditioning protocol; long-term synaptic efficacy changes were measured after approximately 60 minutes. Series resistance was monitored and calculated from the response to a hyperpolarizing potential (−5 mV) step during each sweep throughout the experiments and a variation above 20% led to the rejection of the experiment. Repetitive control stimuli were applied at a frequency of 0.1 Hz for 60 minutes. Drugs were applied in the bath, after recording 10 minutes of baseline and 10 minutes before cellular conditioning protocol, and were present continuously until the end of the recording. Ifenprodil was dissolved in water at 15 mM and then added to extracellular solution for a final concentration of 10 µM (Tocris, Ellisville, MO, USA).

Off-line analysis was performed using Igor-Pro 6.0.3 (Wavemetrics, Lake Oswego, OR, USA). All results are expressed as mean±SEM and statistical significance was assessed using the Student's t-test or the non-parametric Wilcoxon signed-rank test when appropriate at the significance level (p) indicated. Statistical analysis was performed using Prism 5.0 software (San Diego, CA, USA).

### Medium spiny projection neuron model

A dorsal striatum MSPN model cell was created based on the nucleus accumbens neuron model by Wolf et al. (2005) [16]. The Wolf model was translated from NEURON into Genesis simulation software, and channel concentrations and kinetics were adjusted to closely match those of a mature ( >3 weeks old) MSPN in the mouse dorsal striatum (Text S1). The MSPN morphology is the same as in Wolf et al. [16], but with the addition of individual spine compartments on the primary dendrites to the third segment of the tertiary dendrites (Figure 1A). Primary dendrites are 20 µm long, secondary dendrites 24 µm, and each of the 11 tertiary segments is 36 µm long. For all simulations, two adjacent spines were stimulated (on the third segment of the tertiary dendrite, or on the secondary dendrite), and the NMDAR-mediated calcium was recorded from one of the two spines. This model is available on Model DB: http://senselab.med.yale.edu/modeldb/

### Ionic channels

All channels kinetics (Table 1) were taken from published data, using dorsal striatum MSPNs when possible. The Na^+^ kinetics were obtained from dissociated dorsal striatum MSPNs in the guinea pig [59]. The fast A-type potassium channel (Kv4.2) data was obtained from slice dorsal striatum MSPNs in rat [60]. The slow A-type potassium channel (Kv1.2) data was obtained from dissociated and slice dorsal striatum MSPNs in rat [61]. The inwardly rectifying potassium channel (Kir) kinetics were extracted from the computational studies of Wolf et al. [16] and Steephen and Manchanda [62]. The KDr channel was from Migliore et al. [63] The BK channel [64] and SK channel [65] were activated by a specific pool of calcium from the N and R type calcium channels, but not the T or L type calcium channels [16], [66]. L (Cav1.2 and Cav1.3), N, R, and T type voltage sensitive calcium channels use the same parameters as Wolf et al. [16]. Calcium channels were added to the soma, and dendritic shafts. L, R, and T-type channels were also added to spines [20]. MSPNs of the dorsal striatum display different characteristics from those of the ventral striatum [67]. Both hand tuning and the simulated annealing parameter optimization routine in Genesis were used to adjust channel maximal conductances (Table 2), and channel activation and inactivation time constants (Table 1, scaling factor). These parameters were adjusted to match spike frequency, spike width, and latency to first spike extracted from current clamp data obtained at 30–32°C from mouse dorsal striatum MSPN (Figures 1B–D). The change in channel time constants of NaF, KDr, and KAf (scale in table 1) was required to produce the correct spike width as faster time constants produced spikes that were too narrow. In contrast, varying the maximal conductances by ±10% did not significantly change spike width (data not shown). Δt for both experiments and the model MSPN is defined as the time from pre-synaptic stimulation (stimulus artifact in the experimental case) to the peak of the AP.

### Synaptic channels

AMPA and NMDAR channels were added using the *synchan* object in Genesis to all spines in the model. The *synchan* object uses equation 1 to calculate the conductance of the channel from the activation and inactivation time constants (τ_1_ and τ_2_ respectively), time t relative to the action potential, and the maximal conductance (g_max_). K is a normalization constant which is calculated from the time constants and allows G_syn_ to reach a peak value of g_max_.
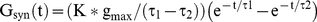
(1)GABA channels were not added to this model, thus all simulations should be interpreted as occurring in the presence of GABA receptor antagonists. The default AMPA maximal conductance is 342 pS, which agrees with data from Carter and Sabatini [20]. Default NMDA maximal conductance was 940 pS to maintain the NMDA/AMPA ratio of 2.75/1 in cortico-striatal terminals [25]. AMPARs have an activation time constant (τ_1_) of 1.1 ms and an inactivation time constant (τ_2_) of 5.75 ms [16], [68]. NMDARs have an activation time constant (τ_1_) 2.25 ms [13], but inactivation (τ_2_) depends on subunit. Magnesium sensitivity to the NMDAR was implemented by using the “Mg_block” object in Genesis which utilizes equation 2:
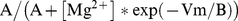
(2)


In which, parameter B = 1/62, while parameter A depends on subunit (Table 3).

Specific GluN2-subunit containing NMDARs were modeled by adjusting the decay time constant, the maximal conductance, and the sensitivity to magnesium block according to published data [23], [24]. GluN2 subunits differ in open probability [69] and affinity for glutamate [70]. These differences, though not modeled explicitly, contribute to the maximal conductance and the decay time which are taken into account in this model. Single decay time constants, averaged from the fast and slow time constants in Vicini et al. [24], were used for each GluN2 subunit (Table 3). All NMDA decay time constants are adjusted for temperature by dividing by a scaling factor of 2. Tau decay in tables 3 and 4 are the scaled values. Maximal conductances were calculated from the slope of the magnesium-free data [23] The ratio between the maximal conductance of GluN2A+B, C, and D was maintained, but the conductances were universally reduced such that the value for GluN2A+B (the predominate subunits in striatal MSPNs) matched cortico-striatal NMDAR/AMPAR ratios [25]. Current-voltage curves for each subunit in the presence of 1 mM magnesium from Monyer et al. [23] were matched by adjusting parameter “A” in this equation (Table 3, Figure S2B).

## Supporting Information

Figure S1 **Added variability to spike time in model MSPN.** Adding low level synaptic input to spines added jitter to the spike time during the 30 ms STDP protocol, but did not alter the shape of the STDP curve. Red circles are the means of 6 jitter trials averaged with the control trial for a total n = 7. Black line is control trial (same trace as green line in figure 2C). Error bars ±SD.
(TIF)

Figure S2 **Characteristics of GluN2 containing NMDARs.** **A**. Model NMDAR current in response to synaptic stimulation in a magnesium-containing (left) and a magnesium-free (right) condition for each GluN2 subunit-containing receptor. **B**. Current-Voltage relationships for each GluN2 subunit-containing NMDAR in the magnesium-free condition (open symbols) and the magnesium-containing condition (filled symbols). Fourth panel shows subunit-specific current-voltage curves overlaid for the magnesium-containing condition only.
(TIF)

Text S1 **Supplemental Methods.** Electrophysiological methods used for tuning the model MSPN.
(DOC)
