# Supplementary figures and images for: The Effects of NMDA Subunit Composition on Calcium Influx and Spike Timing-Dependent Plasticity in Striatal Medium Spiny Neurons
Source: PLoS Comput Biol. 2012 Apr 19;8(4):e1002493. doi: 10.1371/journal.pcbi.1002493 (PMC3334887; doi:10.1371/journal.pcbi.1002493)

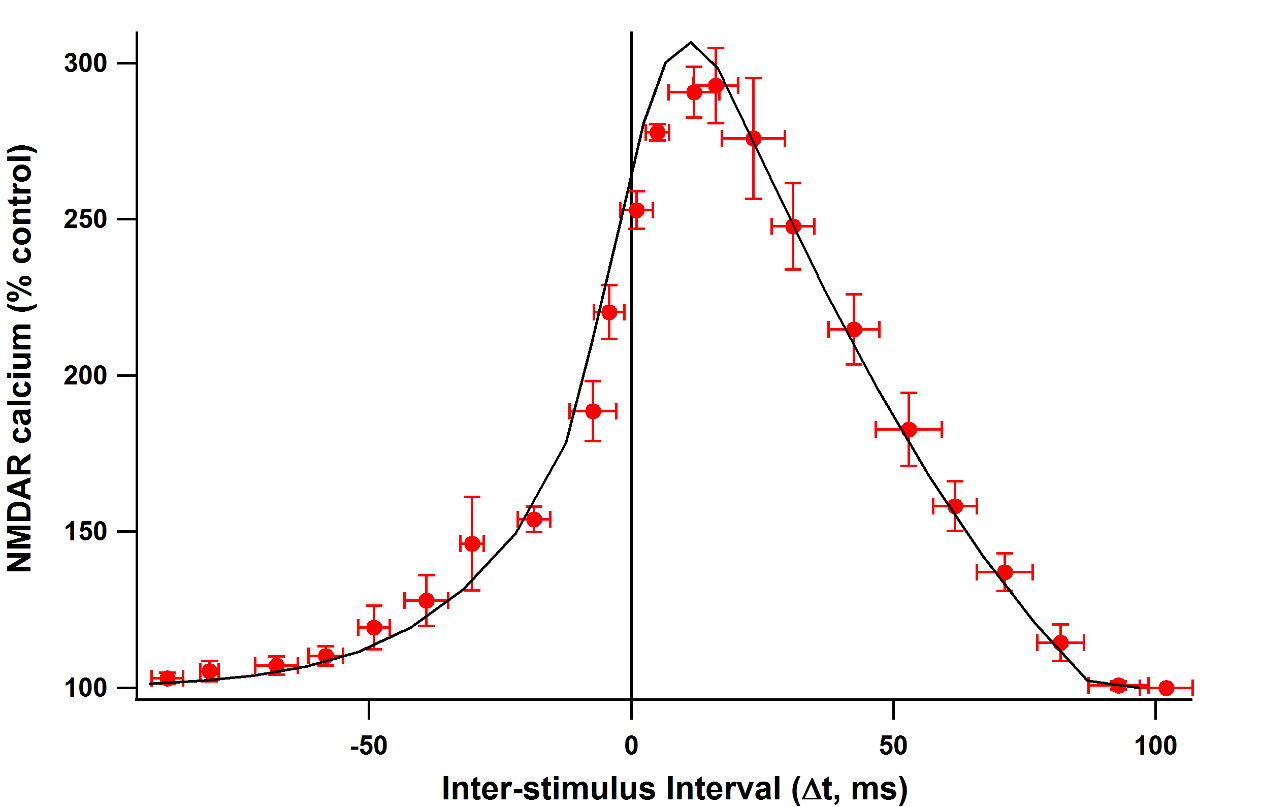

Supplement: Figure S1 — Added variability to spike time in model MSPN. Adding low level synaptic input to spines added jitter to the spike time during the 30 ms STDP protocol, but did not alter the shape of the STDP curve. Red circles are the means of 6 jitter trials averaged with the control trial for a total n = 7. Black line is control trial (same trace as green line in figure 2C). Error bars ±SD. (TIF) [file pcbi.1002493.s001.tif]

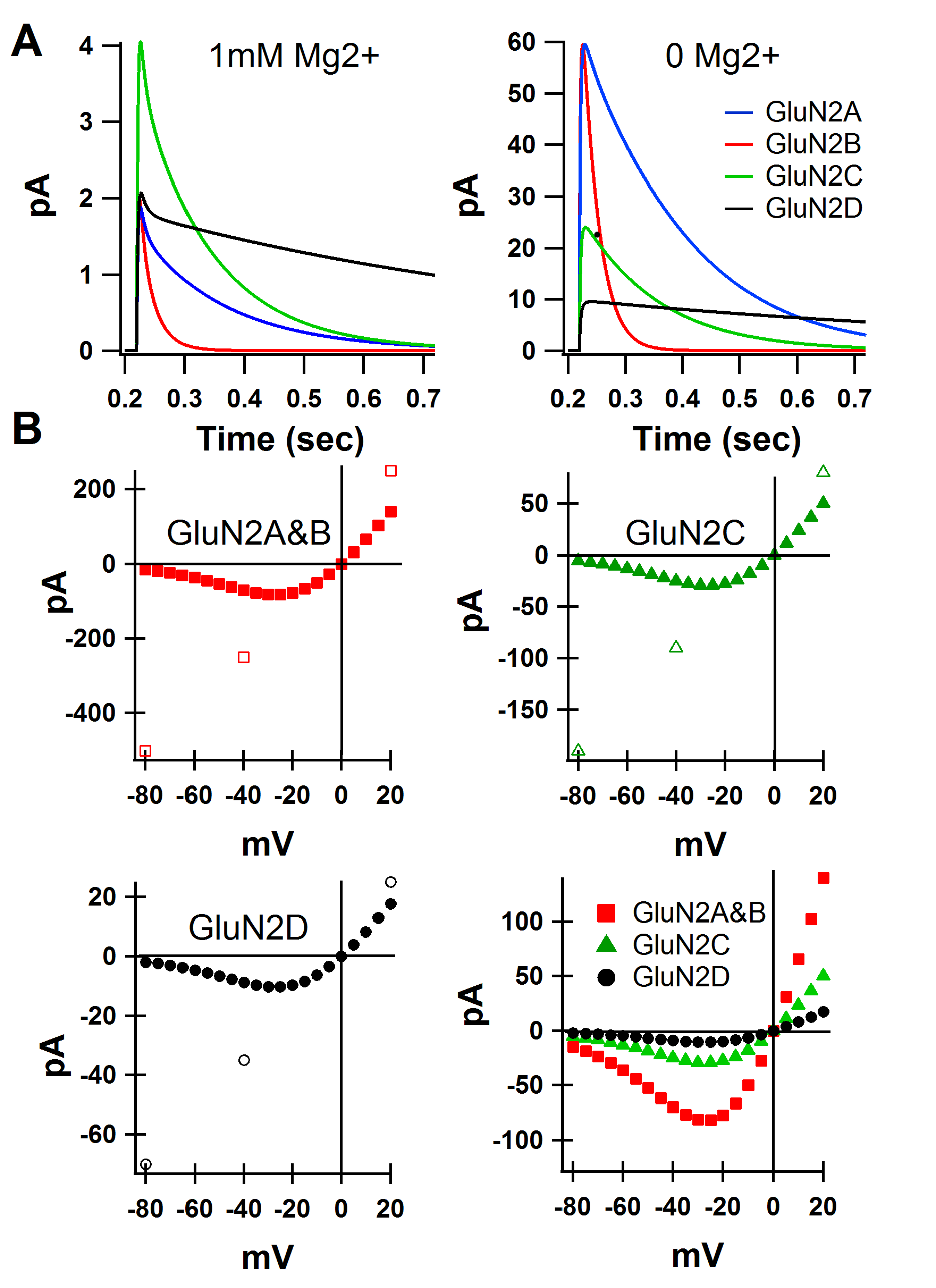

Supplement: Figure S2 — Characteristics of GluN2 containing NMDARs. A. Model NMDAR current in response to synaptic stimulation in a magnesium-containing (left) and a magnesium-free (right) condition for each GluN2 subunit-containing receptor. B. Current-Voltage relationships for each GluN2 subunit-containing NMDAR in the magnesium-free condition (open symbols) and the magnesium-containing condition (filled symbols). Fourth panel shows subunit-specific current-voltage curves overlaid for the magnesium-containing condition only. (TIF) [file pcbi.1002493.s002.tif]
