# Supplementary material for: The Effects of NMDA Subunit Composition on Calcium Influx and Spike Timing-Dependent Plasticity in Striatal Medium Spiny Neurons
Source: PLoS Comput Biol. 2012 Apr 19;8(4):e1002493. doi: 10.1371/journal.pcbi.1002493 (PMC3334887; doi:10.1371/journal.pcbi.1002493)
Supplement: Text S1 — Supplemental Methods. Electrophysiological methods used for tuning the model MSPN. (DOC) [file pcbi.1002493.s003.doc]

**Text S1: Supplemental Methods**

*Electrophysiology for Model Tuning*

C57B6 male and female mice (p20-33) were anesthetized with isoflurane and decapitated. Brains were quickly extracted and placed in oxygenated ice-cold slicing solution (in mM: KCL 2.8, Dextrose 10, NaHCO3 26.2, NaH2PO4 1.25, CaCl 0.5, Mg2SO4 7, Sucrose 210). Hemicoronal slices from both hemispheres were cut 350µm thick using a vibratome (Leica VT 1000S). Slices were immediately placed in an incubation chamber containing artificial cerebrospinal fluid (aCSF) (in mM: NaCl 126, NaH2PO4 1.25, KCl 2.8,CaCl 2, Mg2SO4 1, NaHCO3 26.2, Dextrose 11) for 30 minutes at 33°C, then removed to room temperature (21-24°C) for at least 90 more minutes before use.

A single hemislice was transferred to a submersion recording chamber (ALA Science) gravity-perfused with oxygenated aCSF containing 50µM picrotoxin. Temperature was maintained at 30-32°C (ALA Science) and was monitored with an external thermister. Whole cell patch clamp recordings were obtained from neurons under visual guidance using infrared differential interference contrast imaging (Zeiss Axioskop2 FS plus). Pipettes were pulled from borosilicate glass on a laser pipette puller (Sutter P-2000) and fire-polished (Narishige MF-830) to a resistance of 3-7 MΩ. Pipettes were filled with a potassium based internal solution (in mM: K-gluconate 132, KCl 10, NaCl 8, HEPES 10, Mg-ATP 3.56, Na-GTP 0.38, EGTA 0.1, Biocytin 0.77) for all recordings. Intracellular signals were collected in current clamp and filtered at 3kHz using an Axon2B amplifier (Axon instruments), and sampled at 10-20 kHz using an ITC-16 (Instrutech) and Pulse v8.80 (HEKA Electronik). Series resistance (6-30MΩ) was manually compensated.
